# Chemical Compounds Emitted from *Mentha spicata* Repel *Aromia bungii* Females

**DOI:** 10.3390/insects13030244

**Published:** 2022-02-28

**Authors:** Dandan Cao, Jianfeng Liu, Zhengping Zhao, Xuewu Yan, Weichao Wang, Jianrong Wei

**Affiliations:** 1 Hebei Innovation Center for Bioengineering and Biotechnology, Hebei University, Baoding 071002, China; dandancao@hbu.edu.cn; 2 School of Life Sciences, Hebei University, Baoding 071002, China; jianfengliu@hbu.edu.cn (J.L.); wangweichao@ioz.ac.cn (W.W.); 3 Hunan Academy of Forestry, Changsha 410004, China; zhaozhengping@hnlky.cn (Z.Z.); sbs@hnlky.cn (X.Y.)

**Keywords:** *Aromia bungii*, *Mentha spicata*, behavioural responses, repellent, wood borer

## Abstract

**Simple Summary:**

*Aromia bungii* (Faldermann) (Coleoptera: Cerambycidae) is a serious wood borer of stone fruit trees. Native to parts of Asia and Russia, this beetle recently invaded Germany, Italy, and Japan, causing enormous economic losses. Repellents are effective and sustainable control methods of insect pests. In this study, we identified the *A. bungii* female-repellency ingredients from *Mentha spicata*: myrcene, (*S*)-(+)-carvone, (*E*)-β-caryophyllene, and borneol, as well as their recommended quantities for use. These results contribute to research on repellents that prevent infestation and damage caused by *A. bungii*.

**Abstract:**

*Aromia bungii* (Coleoptera: Cerambycidae) is an economically important wood-boring insect pest of stone fruit trees, particularly *Prunus persica*, in China. It has entered Japan and several European countries as an invasive species in recent years. It is difficult to control because of the cryptic feeding behaviour of larvae beneath the bark. Identification of repellent constituents from non-host plants has potential for use in management strategies against this beetle. *Mentha spicata* is cultivated extensively in Hebei Province (China) as a medicinal plant. Firstly, antennal responses of female *A. bungii* to *M. spicata* volatiles were evaluated by coupled gas chromatography-electroantennograms (GC-EAD), and then the EAD-active components were tested in semi-field trials. The results showed that *A. bungii* females were significantly repelled by myrcene, (*S*)-(+)-carvone, (*E*)-β-caryophyllene, and borneol compared with the control. The presence of myrcene (100 µL; 90% purity), (*S*)-(+)-carvone (200 µL; 96% purity), (*E*)-β-caryophyllene (500 µL; 98.5% purity), and borneol (800 µL; 80% purity) significantly reduced the perching rates of *A. bungii* females on both peach logs and leaves. Considering cost and commercial availability, we suggest that myrcene, (*S*)-(+)-carvone, and (*E*)-β-caryophyllene could be promising repellents against *A. bungii* females in the field.

## 1. Introduction

The red-necked longhorn beetle *Aromia bungii* (Faldermann) (Coleoptera: Cerambycidae: Cerambycinae) is a destructive wood-boring pest of trees in the genus *Prunus*, which includes a number of economically important stone fruit species including peaches, cherries, plums, and apricots [1,2]. This beetle is widely distributed in China, Korea, Mongolia, and eastern Russia [3]. It has invaded Japan, Germany, and Italy, and has been intercepted in cargoes entering the UK and the USA [1]. In 2014, *A. bungii* was added to the EPPO A1 list of pests recommended for regulation as quarantine pests [4,5]. A very recent datasheet from CABI (Center for Agriculture and Bioscience International) stated that *A. bungii* “presents a significant risk to all stone fruit-growing countries in Europe and neighbouring countries” [6].

*Aromia bungii* adults lay eggs in cracks and crevices in the bark of host trees. Developing larvae bore galleries in the phloem and xylem beneath the bark; the complete gallery can reach 50–60 cm in length [7]. Beetles overwinter as larvae and then emerge as adults between June and August. The life cycle lasts 2 to 4 years, depending on the latitude and the climate [8]. The ratio of males to females is about 1:1 [9] and adults can live for over 40 days in the laboratory [10]. Females can mate and oviposit multiple times on the tree trunk, laying an average of >300 eggs [11]. Due to the cryptic feeding damage of larvae and the high fecundity of adults, it has already caused heavy economic losses to *Prunus persica* orchards in China. Since the adult stage coincides with the maturing and harvesting period of peach fruit, it is not recommended to control adults using insecticides. To date, some biological control studies have been done [12,13], and pheromone-based monitoring and control techniques have been developed [14,15,16,17,18,19]. However, *A. bungii* remains a big problem in China.

Plant derivatives or botanical repellents have been used against arthropods for at least two millennia in ancient China, Egypt and India [20,21,22,23,24,25]. For example, *Mentha spicata* (spearmint) has long been used as a medicinal and aromatic plant for its distinctive smell, which makes it very popular as a flavouring and calming agent [26]. Extracts of *M. spicata* stems and leaves are repellent to adult *Plutella xylostella* (Lepidoptera: Plutellidae) [27]; the mosquito, *Anopheles stephensi* (Diptera: Anophelinae) [28]; the carmine spider mite, *Tetranychus cinnabarinus* (Acarina: Tetranychidae) [29,30] and *Frankliniella occidentalis* (Thysanoptera: Thripidae) [31,32]. Since *A. bungii* adults mainly oviposit on the lower sections of peach tree trunks in the orchard, prevention of oviposition could be an operable and effective method for reducing the population density of the next generation of *A. bungii*.

To our knowledge, there is little information concerning the behavioural effect of *M. spicata* on long-horned beetles. We hypothesise that extracts of *M. spicata* or *M. spicata* itself could influence the behaviour of *A. bungii* adults. Firstly, we used gas chromatography-electroantennogram detection (GC-EAD) to screen for active compounds in *M. spicata* volatiles. Secondly, semi-field cage bioassays were conducted to explore what kind of adult behavioural response was aroused by different quantities of EAD-active components. Finally, we used semi-field cage bioassays to check whether *A. bungii* females were repelled by the identified active components from *M. spicata* when in the presence of *P. persica* leaves and stems. We hypothesised that compounds from *M. spicata* that repelled *A. bungii* females could be useful in integrated management strategies.

## 2. Materials and Methods

### 2.1. Insect Collection 

*Prunus persica* trees heavily infested with *A. bungii* larvae were felled and logs transferred from peach orchards in Shunping County, Hebei province, China, to our laboratory in early April 2019. The logs varied in length from 80–120 cm and in diameter from 20–30 cm. Cut edges were sealed with paraffin wax and a plastic film to avoid desiccation, and placed in steel gauze mesh cages (20 mesh sieve) in the laboratory at 25 ± 4 °C, RH 60 ± 10%. Adult *A. bungii* were collected as soon as they emerged in late May to July 2019. Each adult was kept individually in a plastic chamber (PE, 18 cm × 11 cm × 8 cm) and fed with commercial *P. persica* jelly. Adults used for GC-EAD experiments were all more than 3 days old and unmated. Since our main aim was to find the oviposition repellent, only females were tested in our experiments. Females used in the semi-field cage bioassays were captured from peach orchards in Shunping County, Hebei province, China.

### 2.2. Coupled Gas Chromatography-Electroantennogram Detection (GC-EAD)

#### 2.2.1. Collection of Volatiles 

Potted *M. spicata* were purchased from the flower market. Volatiles were collected from 2.7 g *M. spicata* leaves (cut into 1–2 cm pieces: in order to get a high concentration of volatiles) using Tenax-TA adsorbent (60–80 mesh, 100 mg, Sigma-Aldrich, Saint Louis, MO, USA) held in a glass tube (12 cm long with an inner diameter of 0.5 cm), with the Teflon tube connected to the clean air (filtered with colour changing silicone and activated carbon) outlet. Leaf volatiles were collected for 2 h at 400 mL/min flow rate during daylight at 26 °C. Extracted volatiles were eluted with re-steaming chromatographic grade hexane (400 µL), and kept at −20 °C prior to analyses. In total, ten aeration extracts were prepared from *M. spicata* leaves.

#### 2.2.2. GC-EAD Analysis of Volatiles

The *Mentha spicata* aeration extract was analyzed by GC-EAD on an Agilent 7890A GC (Agilent, Santa Clara, CA, USA) fitted with a HP-5 capillary column (30 m × 0.32 mm × 0.25 µm). Nitrogen (99.999% purity) was used as the carrier gas. The injector temperature was 250 °C, and injections were made in splitless mode. The GC oven was programmed from 40 °C for 1 min, then run to 120 °C (held for 1 min) at 10 °C/min, and run to 280 °C (held for 2 min) at 20 °C/min. Column effluent was split equally between the flame ionization detector (FID) and the electroantennogram detector (EAD) with a press-fit Y splitter (Agilent, part number: 5181-3398).

The two terminal flagella of an antenna from an *A. bungii* female were gently cut, then approximately 1 mm from the antennal tip was removed. The prepared antenna was centered in the effluent air stream from the GC. The antennal signals were amplified and filtered using an IDAC-4 data acquisition controller (Syntech, Hilversum, The Netherlands). Data were recorded in parallel with the FID signal and analyzed by GC-EAD 2000 software. In total, analyses were replicated with antennae from 10 females, and 1 antenna was used per female, with each antennal preparation being reused in 2–3 analyses.

### 2.3. GC-MS Analysis of Volatiles

GC-MS was performed with an Agilent Technologies 7890B GC coupled with an Agilent Technologies 5977A mass spectrometer. Samples were analyzed on a HP-5MS UI column (30 m × 0.25 mm × 0.25 µm) (Agilent) to obtain the molecular ion signals, and compared with the NIST 14.0 library. The GC oven conditions were the same as the conditions of the GC-EAD. Helium (99.999% purity) was used as the carrier gas at a rate of 1.2 mL/min. The mass spectrometer was operated in electron-impact (EI) mode (70 eV). The scan range was *m/z* 50–550. The temperatures at ionization source and interface were 230 °C and 285 °C, respectively. Authentic compounds were purchased to compare the retention time (RT) and molecular ion signals with the different chemicals in the volatiles.

### 2.4. Olfactory Response of A. bungii Females to Four Synthetic Chemicals 

#### 2.4.1. Compounds Used

Myrcene (90%) and (*E*)-β-caryophyllene (98.5%) were purchased from Sigma-Aldrich (Saint Louis, MO, USA). Borneol (1000 µg/mL in methanol, including 200 µg/mL isoborneol) was purchased from Tokyo Chemical Industry Co., Ltd. (Tokyo, Japan). (*S*)-(+)-Carvone (96%) was purchased from J&K Scientific Ltd. (Beijing, China). Synthetic chemicals used in bioassays were standard reagents with no dilution.

#### 2.4.2. Semi-Field Cage Bioassays of *A. bungii* Females’ Responses to Synthetic Chemicals

To be closer to the natural environment, two semi-field experiments were conducted in cages (120 cm × 50 cm × 50 cm, Nylon mesh with 60 mesh) under the shadow of trees in the peach orchard in Shunping County, Hebei province, China, in June 2020.

First, different quantities of the four tested synthetic chemicals and distilled water were placed in the two opposite corners of the cage, respectively. For the treatment compounds, the starting quantity was 200 µL, followed by lower quantities (100 µL, 50 µL) or higher quantities (500 µL, 800 µL) to determine the quantities needed to achieve biological activity.

To further test the repellent effects of synthetic chemicals, a second experiment was done using particular chemicals in the presence of the host plant. Host plants were placed in the two opposite corners of each cage and the synthetic chemicals under evaluation placed on one of those host plants. Two different host plant treatments were used: either leaves (15 g) or logs (40 cm in height, 18 cm in diameter) of *P. persica* and the cut edges were sealed with paraffin wax. The synthetic chemicals were presented in polythene-sealed bags (3 cm × 3 cm) containing a particular quantity and were hung on the *P. persica* leaves or logs.

In all tests, one female *A. bungii* was introduced at the central point of each cage. During the bioassay, if the beetle moved to within 30 cm of an odour source in 10 min, it was considered to have made a choice. The position of the two treatments was reversed after every five beetles tested. Each beetle was used only once, and at least 30 responsive adults were required for each choice test. All bioassays were conducted from 8:30 to 10:00 AM beneath the natural light at 23 ± 2 °C, and the RH was 70 ± 5%.

### 2.5. Statistical Analyses

Data for behavioural responses in the semi-field cage tests were dependent and did not have a normal distribution, so the binomial test (Nonparametric tests) was used to identify significant differences in the responses of *A. bungii* adults to different odours in the semi-field cage tests. There were at least 30 adults that made choices in each experiment. All analyses were conducted by SPSS software (Version, 22.0).

## 3. Results

### 3.1. Identification of Active Chemicals from M. spicata Volatiles 

**Figure 1:**
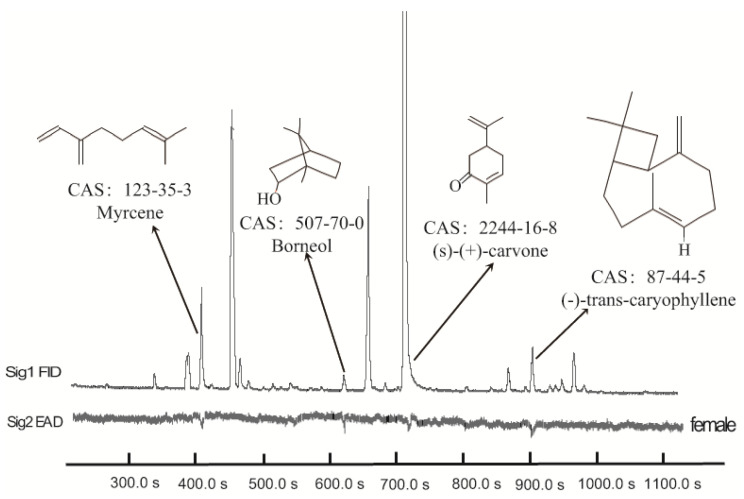
Coupled gas chromatography-electroantennogram detection of *M. spicata* volatiles. The upper trace is the GC chromatogram; the lower inverted trace is the antennal response of a female *A. bungii*; CAS number, chemical names, and structural formulae of antennal-active compounds are indicated.

In GC-EAD analysis of *M. spicata* volatiles, antennae of female *A. bungii* responded to four components (Figure 1), which were then identified by GC-MS as myrcene (*m/z* 136, *m/z* 93, *m/z* 79, *m/z* 69), borneol (*m/z* 154, *m/z* 110, *m/z* 95, *m/z* 67), (*S*)-(+)-carvone (*m/z* 150, *m/z* 108, *m/z* 82, *m/z* 54), and (*E*)-β-caryophyllene (*m/z* 204, *m/z* 133, *m/z* 93, *m/z* 79), respectively. They were confirmed again with those of authentic myrcene, borneol, (*S*)-(+)-carvone, and (*E*)-β-caryophyllene.

### 3.2. Semi-Field Cage Bioassay of A. bungii Females to Different Quantities of Four Synthetic Chemicals 

**Figure 2:**
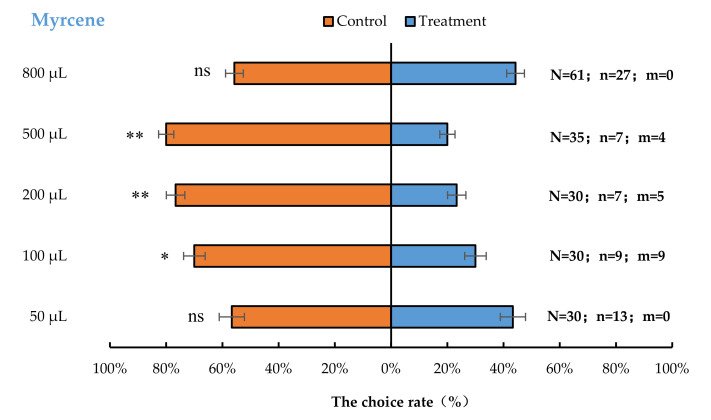
Behavioural responses of *A. bungii* females to different quantities of myrcene. Note: N = number of responsive *A. bungii*; n = number of *A. bungii* females choosing the treatment; m = number of *A. bungii* that made no choice. ns = no significant difference; * = significant difference at the 0.05 level. ** = significant difference at the 0.01 level (the binomial test).

The result of the cage bioassays showed that *A. bungii* females exhibited significant behavioural repulsion to 100 µL myrcene (*p* < 0.05), 200 µL myrcene (*p* < 0.01), and 500 µL myrcene (*p* < 0.01), but no significant behavioural response to 50 µL (*p* = 0.59) or 800 µL (*p* = 0.37) myrcene compared with the control (Figure 2).

**Figure 3:**
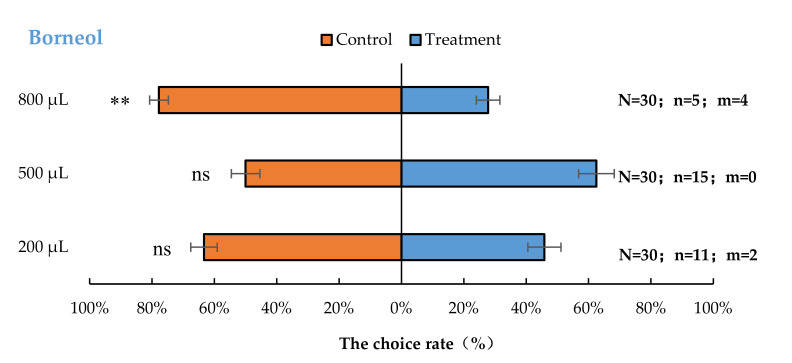
Behavioural responses of *A. bungii* females to different quantities of borneol. Note: N = number of responsive *A. bungii*; n = number of *A. bungii* females choosing the treatment; m = number of *A. bungii* that made no choice. ns = no significant difference; ** = significant difference at the 0.01 level (the binomial test).

*Aromia bungii* females exhibited significant behavioural repulsion to 800 µL borneol (*p* < 0.01), but no significant behavioural response to 200 µL (*p* = 0.20) and 500 µL (*p* = 1.00) borneol compared with the control (Figure 3).

**Figure 4:**
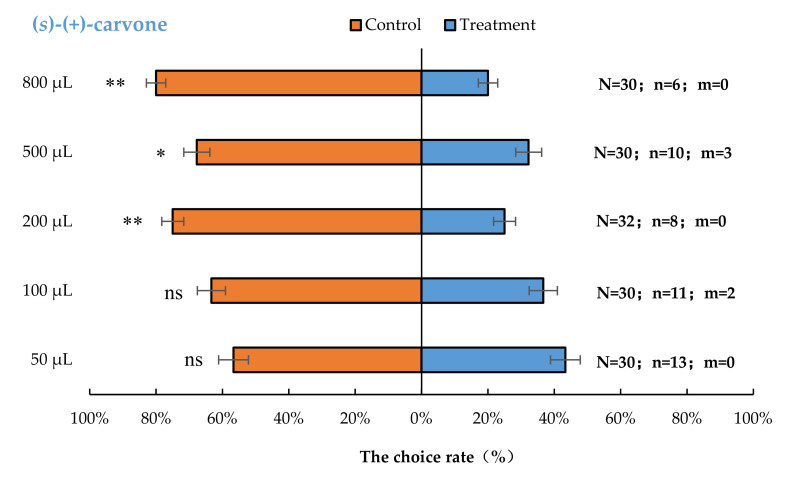
Behavioural responses of *A. bungii* females to different quantities of (*S*)-(+)-carvone. Note: N = number of responsive *A. bungii*; n = number of *A. bungii* females choosing the treatment; m = number of *A. bungii* that made no choice. ns = no significant difference; * = significant difference at the 0.05 level. ** = significant difference at the 0.01 level (the binomial test).

*Aromia bungii* females exhibited significant behavioural repulsion to 500 µL (*S*)-(+)-carvone (*p* < 0.05), 200 µL (*S*)-(+)-carvone (*p* < 0.01), and 800 µL (*S*)-(+)-carvone (*p* < 0.01), but no significant behavioural response to 50 µL (*p* = 0.59) and 100 µL (*p* = 0.20) (*S*)-(+)-carvone compared with the control (Figure 4).

**Figure 5:**
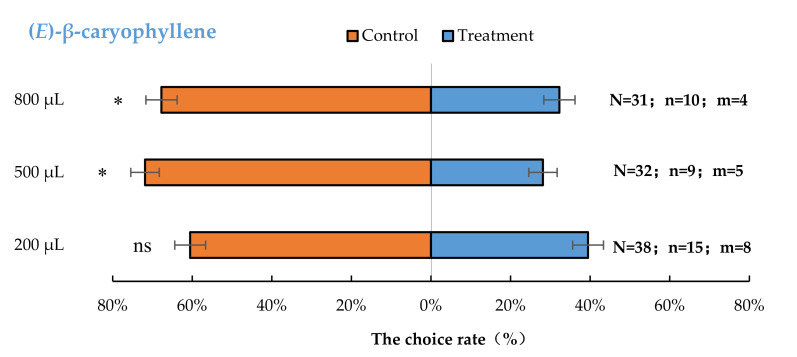
Behavioural responses of *A. bungii* females to different quantities of (*E*)-β-caryophyllene. Note: N = number of responsive *A. bungii*; n = number of *A. bungii* females choosing the treatment; m = number of *A. bungii* that made no choice. ns = no significant difference; * = significant difference at the 0.05 level.

*Aromia bungii* females exhibited significant behavioural repulsion to 500 µL (*E*)-β-caryophyllene (*p* < 0.05) and 800 µL (*E*)-β-caryophyllene (*p* < 0.05), but no significant behavioural response to 200 µL (*p* = 0.26) (*E*)-β-caryophyllene compared with the control (Figure 5).

### 3.3. Semi-Field Cage Bioassay of A. bungii Females’ Responses to Host Plant in the Presence of Synthetic Chemicals 

**Figure 6:**
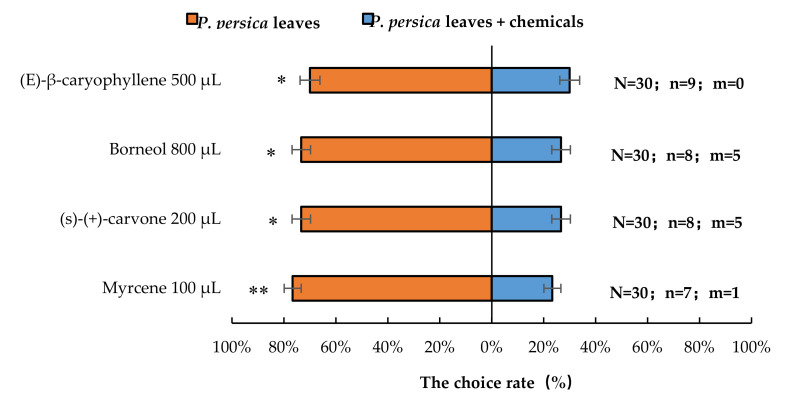
Behavioural responses of *A. bungii* females to *P. persica* leaves in the presence of synthetic chemicals. Note: N = number of responsive *A. bungii*; n = number of *A. bungii* females choosing the treatment; m = number of *A. bungii* that made no choice. * = significant difference at the 0.05 level. ** = significant difference at the 0.01 level (the binomial test).

The results showed that the presence of 200 µL (*S*)-(+)-carvone (*p* < 0.05), 500 µL (*E*)-β-caryophyllene (*p* < 0.05), 100 µL myrcene (*p* < 0.01), or 800 µL borneol (*p* < 0.05) significantly reduced the number of *A. bungii* females choosing host plant *P. persica* leaves (Figure 6).

**Figure 7:**
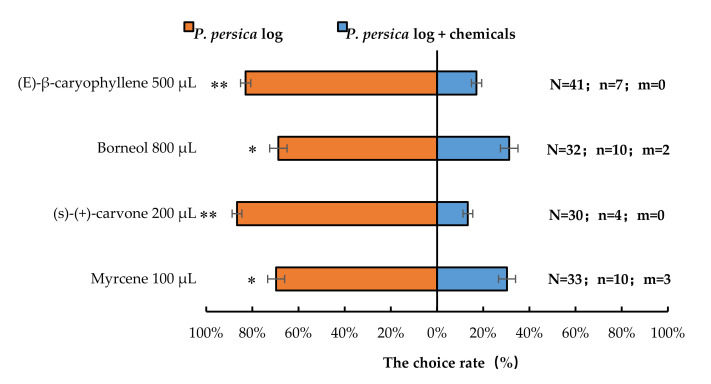
Behavioural responses of *A. bungii* females to *P. persica* logs in the presence of synthetic chemicals. Note: N = number of responsive *A. bungii*; n = number of *A. bungii* females choosing the treatment; m = number of *A. bungii* that made no choice. * = significant difference at the 0.05 level. ** = significant difference at the 0.01 level (the binomial test).

Similarly, the presence of 100 µL myrcene (*p* < 0.05), 800 µL borneol (*p* < 0.05), 200 µL (*S*)-(+)-carvone (*p* < 0.01), or 500 µL (*E*)-β-caryophyllene (*p* < 0.01) significantly reduced the number of *A. bungii* females choosing host *P. persica* logs. Therefore, compared with *P. persica* leaves or logs alone, *P. persica* leaves or logs in presence with synthetic chemicals significantly repelled *A. bungii* females, respectively (Figure 7).

## 4. Discussion

Some compounds derived from *M. spicata* essential oils and plant extracts have exhibited insecticidal and insect-repellent activity for mosquitoes and stored products pests [33]. Therefore, we extracted the volatiles from *M. spicata* in order to identify the active components with respect to *A. bungii* adults, and four EAD-active components were obtained.

Some studies have shown that myrcene is an aggregation synergist [34] for *Monochamus alternatus* (Coleoptera: Cerambycidae) [35] and *Megalurothrips sjostedti* (Thysanoptera: Thripidae) [36,37]. However, *A. bungii* females showed significant repulsion to 100 µL, 200 µL, and 500 µL myrcene in the semi-field cage bioassays, and 100 µL myrcene significantly reduced the number of *A. bungii* females choosing the host plant. The olfactory organ of *A. bungii* might have become desensitized in the high quantity of myrcene (800 µL) treatment in cages. Whether myrcene has a synergistic effect on other repellent chemicals will be tested in a future study.

The aroma and flavour of *M. spicata* is mainly due to the presence of carvone [38]. Carvone is a major contributor to the fumigant activity, contact toxicity and antifeedant activity of *M. spicata* against *Blattella germanica* (Blattodea: Blmtdlidae) [39], *Deroceras reticulatum* (Gastropoda: Pulmonata: Stylommatophora) [40], *Meloidogyne incognita* (Tylenchida: Meloidogynidae), and *Hylobius abieties* (Coleoptera: Curculionidae) [41,42,43,44]. As we expected, 200 µL, 500 µL, and 800 µL (*S*)-(+)-carvone all showed a clear repellent effect on *A. bungii* females in our study.

(*E*)-β-caryophyllene is a chemical component of many plants, and has been reported as an insect repellant [45]. For instance, it was repellent to *Reticulitermes flaviceps* (Isoptera: Rhinotermitidae) [46], *Aphis gossypii* Glover (Homoptera: Aphididae) [47], *Aedes aegypti* (Diptera: Culicidae) [48], and *Agrilus zanthoxylumi* (Coleoptera: Buprestidae) [49]. In our study, it was shown to have a repellent effect on *A. bungii* females (500 µL and 800 µL). (*E*)-β-caryophyllene is also attractive to *Coccinella septempunctata* (Coleoptera: Coccinellidae) at a quantity of 20 µg [50], and lower quantities of (*E*)-β-caryophyllene (0.01 µg to 10 µg) are attractive to both virgin and mated *Drosophila suzukii* (Diptera: Drosophilidae) females (0.01 µg), though higher quantities repelled them [51]. Therefore, the influence of (*E*)-β-caryophyllene on insect behaviour might be related to the quantities present.

Borneol is known to attract *Cyzenis albicans* (Diptera: Tachinidae), a tachinid parasitoid of the winter moth, *Operophtera brumata* (Lepidoptera: Geometridae) [52]. However, borneol was significantly repellent to *A. bungii* females at the 800 µL quantity in our study. If commercial costs are considered, then we would not recommend borneol as an *A. bungii* repellent for use in the field.

Deterrence of *A. bungii* oviposition could reduce populations and be useful in management strategies. Myrcene, borneol, (*S*)-(+)-carvone, and (*E*)-β-caryophyllene were all repellent to *A. bungii* females in this study. However, several issues such as customer approval, formulation, and non-target toxicity must be addressed before they can be used in practice [53,54]. They also have potential to be used together with aggregation sex pheromones in the ‘push and pull strategy’ for control of *A. bungii*.

## 5. Conclusions

Our results show that myrcene, borneol, (*S*)-(+)-carvone, and (*E*)-β-caryophyllene from *M. spicata* were all repellent to *A. bungii* females, and the effective quantities were 100 µL, 800 µL, 200 µL, and 500 µL, respectively. Taking commercial costs and quantity into consideration, myrcene, (*S*)-(+)-carvone, (*E*)-β-caryophyllene, or a three-component blend have greatest potential as repellents for management of *A. bungii* females.

## Data Availability

Data can be provided on request from the lead author.
